# Automatic versus Manual Suturing in Total Laryngectomy: Fistula Incidence, Margin Status, and Operative Time

**DOI:** 10.1055/s-0046-1819643

**Published:** 2026-04-30

**Authors:** José Alberto Fernandes, António Andrade, Pedro Valente, Ricardo Vaz, Diogo Pereira, Helena Silveira

**Affiliations:** 1 Department of Otorhinolaryngology, Unidade Local de Saúde de São João, Porto, Portugal.

**Keywords:** total laryngectomy, pharyngocutaneous fistula, automatic suturing; manual suturing, laryngeal carcinoma, pharyngeal reconstruction

## Abstract

**Introduction:**

Pharyngeal reconstruction is essential after total laryngectomy. Traditionally, manual suturing (MS) is used, but it often involves longer operative times and variable outcomes. Over the years, automatic suturing (AS) has gained attention due to its precision, shorter operative time, and lower complication rates.

**Objective:**

To compare AS and MS in patients with laryngeal carcinoma who underwent total laryngectomy, focusing on pharyngocutaneous fistula (PCF) incidence, operative time, surgical margins, and hospital stay.

**Methods:**

A retrospective cohort from 2014 to 2024 at a tertiary center, including 107 patients with laryngeal carcinoma who underwent pharyngeal reconstruction with AS or MS. The suturing choice depended on oncological safety and surgeon preference. Data on demographics, tumor stage, PCF occurrence, hospital stay, and operative time were collected.

**Results:**

A total of 53 patients underwent AS, and 54, MS. Most were male subjects (96.2%), with similar ages (63.3 versus 62.3 years; *p* = 0.559). The PCF rates were higher with AS (26.4 versus 18.5%) but not statistically significant ( *p* = 0.328). No correlation was found between salvage laryngectomy and PCF. Negative margins (R0) were more frequently observed in the AS group (82.1 versus 37%; *p* = 0.04). Operative time was shorter in AS (316.8 versus 367.2 minutes; *p* = 0.01). Hospital stay and time to oral feeding were similar.

**Conclusion:**

There was a noted association between AS and shorter operative time and a higher observed rate of negative margins in selected cases, without an increase in PCF. These findings should be interpreted in the context of selection bias and anatomical constraints.

## Introduction

Total laryngectomy is a well-established surgical procedure for the treatment of advanced or recurrent laryngeal squamous cell carcinoma, offering favorable oncological outcomes and improving patient survival. 1 Despite its therapeutic benefits, this surgery is associated with significant postoperative complications. Among these, pharyngocutaneous fistula (PCF) stands out as the most frequent and challenging, affecting approximately 20 to 30% of patients in various clinical series. 2 The presence of PCF can result in prolonged hospitalization, delayed adjuvant therapy, increased morbidity, and higher healthcare costs, thus representing a considerable clinical burden.

Pharyngeal reconstruction after total laryngectomy is traditionally performed using manual suturing (MS) in three layers—mucosa, submucosa, and muscle—to ensure a robust closure and prevent leakage. However, advances in surgical technology have introduced linear staplers that enable automatic suturing (AS), which may offer several potential advantages, including standardized closure, reduced operative time, more consistent tissue approximation and a lower risk of surgical field contamination by intraluminal contents from the esophageal tract. 3

Preliminary studies suggest that AS can reduce operative time and yield PCF rates comparable to or lower than MS, although evidence remains inconsistent due to methodological and population differences. 4 Additionally, no clear consensus exists regarding the impact of AS on oncological safety margins and postoperative recovery.

## Objective

This study aims to compare AS and MS techniques for pharyngeal reconstruction following total laryngectomy in patients with laryngeal carcinoma. The key outcomes assessed include incidence of PCF, operative time, surgical margin status, duration of hospitalization, and time to initiation of oral feeding.

## Methods

### Study Design

A single-center, retrospective study that included all patients who underwent total laryngectomy with primary pharyngeal reconstruction between January 2014 and December 2024.

### Patient Cohort

Demographic data, tumor staging, postoperative complications (notably PCF), duration of hospitalization, initiation of oral feeding, and operative time were collected.

Patients were divided into two groups according to the method used for pharyngeal defect closure. In all cases, neck dissection was performed according to oncologic indication (bilateral selective/modified radical).

Tumors invading the oropharynx (including the base of the tongue and/or vallecula) or the hypopharynx (piriform sinus) were excluded to ensure safe surgical margins. The use of AS was restricted to tumors confined to the larynx or with limited anterior extralaryngeal extension, after both preoperative imaging and intraoperative assessment confirmed no involvement of the base of tongue, vallecula, or pyriform sinus. These anatomical regions are considered contraindications to AS due to the risk of inadequate oncologic safety. Margin assessment was performed on the resection specimen by the pathology department, according to institutional head and neck protocols.

This study was approved by the Ethics Committee of our institution. All procedures involving human participants were conducted in accordance with the ethical standards of the institutional and/or national research committee, as well as with the 1964 Helsinki Declaration and its later amendments. Given the retrospective nature of this study, the requirement for informed consent was waived by the ethics committee.

## Surgical Techniques

The decision to use AS or MS was primarily guided by two key factors: the need to secure oncological safety margins that would enable complete and effective tumor resection and the surgeon's individual experience. This decision was made pre- and/or intraoperatively, considering the anatomical characteristics of the surgical defect and the extent of the lesion.

In the present study, pharyngeal reconstruction was performed using two distinct techniques. In the AS group, a linear stapler—either Medtronic—was used, with the staple line subsequently reinforced by simple interrupted sutures using Vicryl 3/0 (Ethicon Inc.). In the MS group, a continuous three-layer closure—mucosa, submucosa, and muscle—was performed using inverted stitches following the Connell technique, with Vicryl 3/0. Both techniques were applied based on intraoperative criteria defined by the surgical team, considering the anatomical features of the defect and tumor extension.

## Statistical Analysis

Quantitative variables were described using mean and standard deviation (SD) values, while categorical variables were presented as absolute frequencies and percentages. To compare means between both groups, the independent samples Student's *t* -test was used when the assumption of normality was confirmed by the Shapiro–Wilk test; in cases of non-normal distribution, the Mann–Whitney U test was applied. For the comparison of proportions between categorical variables, the chi-squared test was used; when the expected frequency in any cell was less than 5, Fisher's exact test was applied. A two-sided *p* -value < 0.05 was considered statistically significant.

In addition to these univariate analyses, multivariable analyses were performed to adjust for potential confounding variables and to assess the independent association between surgical technique and study outcomes. Logistic regression models were applied for binary outcomes, namely the occurrence of PCF and the achievement of negative surgical margins (R0), while linear regression models were used for continuous outcomes, including operative time, length of hospital stay, and time to initiation of oral feeding. All models were adjusted for age, sex, tumor stage (T4 versus T3), and type of surgery (primary vs. salvage laryngectomy). Results from the logistic regression analyses were expressed as adjusted odds ratios (ORs) with 95%CIs, and linear regression results were presented as adjusted β coefficients with a corresponding 95%CI. Statistical analyses were performed using the IBM SPSS Statistics for Windows (IBM Corp.), version 26, and Python (Python Software Foundation; statsmodels package) for multivariable modeling.

## Results

### Sample Characterization

A total of 107 patients with laryngeal carcinoma were included in the study, of whom 53 underwent AS and 54 underwent MS. The sample was predominantly composed of male patients (96.2%), and the mean age was similar between the two groups (62.7 ± 8.94 versus 63.0 ± 6.88 years respectively).

### Manual Versus Automatic Suturing

Given PCF's clinical relevance as the most common and potentially serious complication, was the primary outcome of interest. In the present study, the incidence of PCF was 26.4% in the AS group and 18.5% in the MS group, with no statistically significant difference between them ( *p* = 0.328). Both groups included cases of primary and salvage laryngectomy; however, no statistically significant differences in fistula incidence were observed between these subgroups ( Table 1 ).

**Table 1:** Incidence of PCF according to suturing technique (automatic versus manual) and surgical context (primary versus salvage total laryngectomy)

	AS group (n = 53)		MS group (n = 54)		Total (n = 107)	
Salvage	PCF	No PCF	PCF	No PCF	PCF	No PCF
Yes: n (%)	3 (5.66)	0 (0)	0 (0)	0 (0)	3 (2.8)	0 (0)
No: n (%)	11 (20.75)	39 (73.6)	10 (18.5)	44 (81.5)	21 (19.6)	83 (77.6)

**Abbreviations:** AS, automatic suturing; MS, manual suturing; PCF, pharyngocutaneous fistula.

**Fig. 1:**
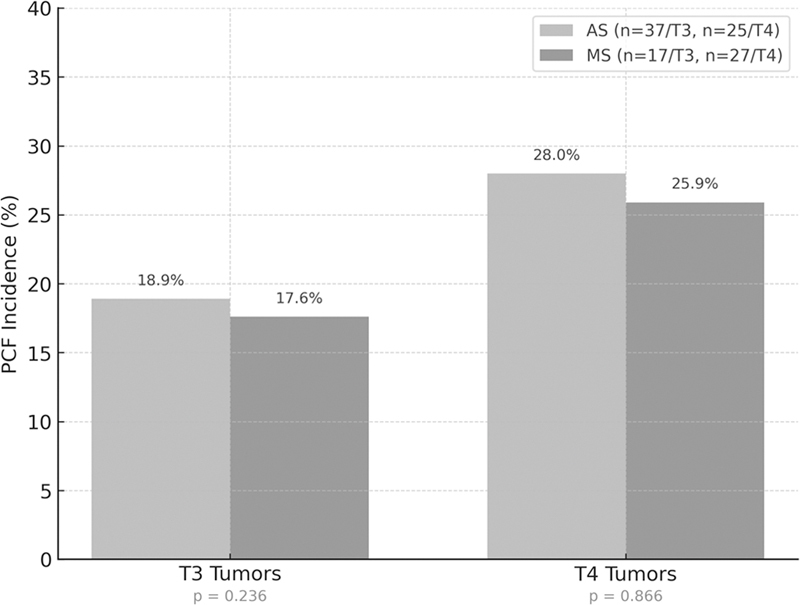
Pharyngocutaneous fistula (PCF) rates by tumor stage. Bars represent the proportion of patients for each stage category.

When stratified by pathological stage (T3 and T4), no significant differences in PCF incidence were found between manual and automatic suturing techniques ( Fig. 1 ). Among patients with T3 tumors, PCF was observed in 7 cases (18.9%) in the AS group and 3 cases (17.6%) in the MS group ( *p* = 0.236). Similarly, for T4 tumors, PCF occurred in 7 patients (28.0%) in the AS group and 7 patients (25.9%) in the MS group ( *p* = 0.866).

A higher proportion of negative surgical margins (R0) was observed in the AS group (82.1%) compared with the MS group (37%; *p* = 0.040). Table 2 compares various surgical details between the groups.

**Table 2:** Comparison of surgical margin status between the AS and MS groups

	AS group (n = 53)	MS group (n = 54)	*p* -value
	Surgical margins		
R0: n (%)	46 (82.1)	20 (37)	0.04
R1: n (%)	7 (13.2)	34 (63)	0.04

**Abbreviations:** AS, automatic suturing; MS, manual suturing.

Operative time was significantly shorter in the AS group (316.8 ± 66.2 minutes) than in the MS group (367.2 ± 83.7 minutes; *p* = 0.01). Although the AS group also showed a shorter mean hospital stay (16.8 ± 5.2 days) and earlier initiation of oral feeding (12.5 ± 4.3 days) compared with the MS group (19.3 ± 6.1 and 16.2 ± 5.7 days respectively), these differences did not reach statistical significance ( *p* = 0.334 and 0.323 respectively). Table 3 presents a comparison of these variables between the two groups.

**Table 3:** Comparison of perioperative outcomes between AS and MS groups

	AS group (n = 53)	MS group (n = 54)	*p* -value
Mean length of hospitalization (days)	16.8 ± 6.35	19.3 ± 10.3	0.334
Mean time until the beginning of oral feeding (days)	12.53 ± 7.43	16.22 ± 13.4	0.323
Mean operative time (minutes)	316.8 ± 66.2	367.2 ± 83.7	0.01

**Abbreviations:** AS, automatic suturing; MS, manual suturing.

**Fig. 2:**
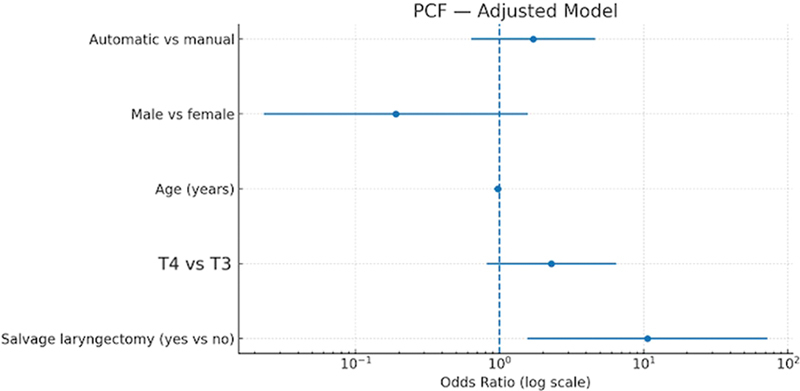
Forest plot showing adjusted odds ratios (log scale) and 95% CIs for predictors of PCF after total laryngectomy.

**Fig. 3:**
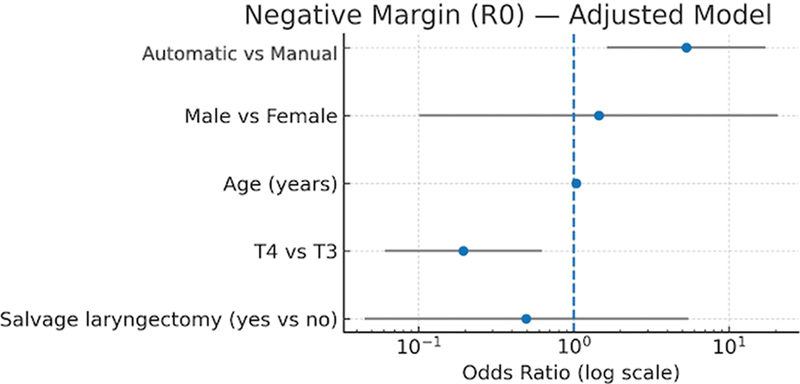
Forest plot showing adjusted odds ratios (log scale) and 95% CIs for predictors of negative surgical margins (R0).

In the adjusted logistic regression model for PCF, none of the evaluated variables—including surgical technique, sex, age, tumor stage, or salvage laryngectomy—were significantly associated with the outcome. The AS group demonstrated an adjusted OR of 1.43 (95%CI, 0.47–4.34; *p* = 0.531) compared with MS, indicating no statistically significant difference in PCF risk after controlling for confounding variables ( Fig. 2 ). Conversely, in the model for achieving negative surgical margins (R0), AS emerged as a strong independent predictor, with an adjusted OR of 5.31 (95%CI, 1.64–17.21; *p* = 0.005), corresponding to more than a 5-fold increase in the odds of obtaining negative margins compared with MS ( Fig. 3 ). No other covariates demonstrated a statistically significant association in either model.

## Discussion

This retrospective study suggests that AS may offer certain surgical advantages over MS in pharyngeal reconstruction following total laryngectomy. The significantly shorter operative time observed in the AS group is consistent with previous reports and is likely related to the technical standardization and efficiency of stapler devices. 4 5 6

Regarding oncologic safety, our findings should not be interpreted as indicating that the closure technique directly affects margin status. The higher frequency of negative margins in the AS group most probably reflects case selection and tumor anatomy, as staplers were limited to midline or anteriorly confined tumors, whereas posterior and supraglottic lesions—known for submucosal spread and difficult exposure—were closed manually. Similar associations have been reported in previous studies 8 7 8 and should be interpreted as a reflection of patient selection rather than a margin-improving effect of the technique. Case selection therefore remains the most plausible explanation, although technical aspects of AS—such as uniform tissue compression, reduced field manipulation, and standardized closure—may also have contributed to consistency in margin status.

In our cohort, most positive margins occurred in the lateroposterior pharyngeal wall and were predominantly associated with supraglottic tumors. These lesions are prone to submucosal microscopic spread that is frequently underestimated during preoperative staging and only becomes evident after resection, making it difficult to achieve widely negative margins even under direct visualization. 8 Furthermore, supraglottic and posteriorly located tumors are anatomically less favorable due to their proximity to the base of the tongue and hypopharynx, where achieving safe clearance is technically challenging. In several cases, intraoperative findings revealed greater extension than anticipated, precluding stapler use and necessitating manual closure.

With respect to PCF, no statistically significant differences were observed between groups in either unadjusted or adjusted analyses. In multivariable regression, AS was not associated with a significant effect on fistula risk after controlling for confounders. This is consistent with prior studies 7 and may reflect unmeasured patient-related risk factors, such as nutritional status, comorbidities, and previous radiotherapy. 9 The relatively small number of salvage laryngectomy cases prevents definitive conclusions, although salvage surgery has consistently been identified as a major risk factor for PCF in meta-analyses. 2

Although shorter hospital stays and earlier initiation of oral feeding were observed in the AS group, these differences did not reach statistical significance after adjustment. This aligns with previous evidence suggesting that standardized closure techniques may support postoperative recovery, 1 10 though our sample size may have limited the detection of modest but clinically relevant effects.

The main limitations of this study include its retrospective design, moderate sample size, and single-center setting. The surgeon-driven selection of closure technique introduces unavoidable bias, and stapling devices not specifically designed for pharyngeal reconstruction may also have influenced technical outcomes. The observed difference in margin status is therefore subject to confounding by indication, as the choice of closure technique was determined by tumor location and exposure. This observation should not be interpreted as evidence that stapling improves resection margins.

Despite these limitations, our findings suggest that AS is a safe and efficient technique for pharyngeal reconstruction, without increasing the risk of PCF. Prospective, multicenter, randomized trials with long-term follow-up are warranted to confirm these results and evaluate functional and quality-of-life outcomes.

## Conclusion

The AS technique appears to be safe and efficient for pharyngeal reconstruction following total laryngectomy, associated with shorter operative time and comparable oncologic safety to MS. Larger prospective studies are needed to confirm these results.
